# Protein folding, protein dynamics and the topology of self-motions

**DOI:** 10.1098/rsos.240873

**Published:** 2024-09-18

**Authors:** Steven Hayward

**Affiliations:** ^1^ Computational Biology Group, School of Computing Sciences, University of East Anglia, Norwich, UK

**Keywords:** protein topology, redundant manipulator, co-regular surface, inverse kinematics

## Abstract

It has long been recognized that segments of the protein main chain are like robotic manipulators and inverse kinematics methods from robotics have been applied to model loops to bridge gaps in protein comparative modelling. The complex internal motion of a redundant manipulator with fixed ends is called a self-motion and its character is determined by the relative position of its ends. Self-motions that are topologically equivalent (homotopic) occupy the same continous region of the configuration space. Topologically inequivalent (non-homotopic) regions are separated by co-regular surfaces and crossing a co-regular surface can result in a sudden dramatic change in the character of the self-motion. It is shown, using a five-residue type I β-turn, that these concepts apply to protein segments and that as the ends of the five-residue segment come closer together, a co-regular surface is crossed, and the structure is locked in to becoming either a type I or type I′ turn. It is also shown that the type II turn is topologically equivalent to the type I′ turn, not the type I turn. These results have implications for both native-state protein dynamics and protein folding.

## 1. Introduction

During the folding of a globular protein, the two ends of a main-chain segment will in general come closer together as a compact structure forms. Changes in the structure of a segment will be mainly driven by external forces acting directly on the segment and the movement of the flanking protein chain. In addition to these external drivers, there are the internal constraints. These constraints, created by the rigid bonds mean that a protein segment does not change conformation like a piece of string but has much more complex dynamics. In this study, it is shown that concepts concerning the topologies of motions of robotic manipulators [1] are applicable to the rigid-bond model of a protein main-chain segment. It is argued that the findings are valid for more realistic models and have implications for protein folding and protein dynamics. In particular, they show how during folding, the character of the internal motion of a segment can change suddenly and dramatically. They also show how it could get locked into forming a particular structure early in the folding process suggesting a link to the folding funnel concept [2].

### 1.1. Robotic manipulator as a model of a protein main-chain segment

Robotic manipulators have rigid links connected by joints that enable relative motion of the links according to the type of joint. A reasonable model of the protein main chain is one where the bond lengths, bond angles and ω-torsions are fixed and the only degrees of freedom are the ϕ , ψ torsions. Thus, a protein main-chain segment can be described as a robotic manipulator with links (the bonds) arranged serially with revolute joints (the ϕ , ψ torsions). A robotic arm will have a base from which the first link extends and at the end of the final link a tool called an ‘end-effector’. Here, we will be concerned with an *n*R manipulator, where *n* is the number of joints or for proteins the number of ϕ , ψ torsions. The process in robotics of determining the joint angles to achieve a desired position and orientation of the end-effector is referred to as ‘inverse kinematics’. Given that an end-effector is a rigid body with six degrees of freedom, in general this requires n≥6 . If n=6 , then generally for a specified position and orientation of the end-effector, the arm will be fixed, i.e. the joint angles are not able to vary. Such a system is ‘non-redundant’. For *n* > 6, even if the end-effector is fixed, the joints will be able to vary. For this ‘redundant’ manipulator the arm can undergo continuous variation, called a ‘self-motion.’ In proteins, self-motions model the motion that a loop undergoes when both its ends are fixed.

In this work, we will be concerned with the topologies of self-motions and how understanding these leads to insights into protein dynamics and folding.

### 1.2. Inverse kinematics methods in protein loop modelling and protein loop dynamics

Inverse kinematics methods have been used in protein research especially for modelling loops to bridge the gap between two fixed end points as part of homology or comparative modelling [3]. Go & Scheraga [4] were the first to find an analytical method to do this but the analogy to the *n*R robotic manipulator described above means that methods from robotics can be applied to this problem. These include cyclic coordinate descent [5] and those methods that reduce the problem to solving a polynomial of degree 16 to find a maximum of 16 different bridging loop conformations [6]. These nonlinear inverse kinematics methods are ideal for loop modelling in comparative modelling scenarios, but there is also another application area which is to model protein dynamics. Use of nonlinear methods for the study of dynamics of a loop with n rotatable bonds where *n* > 6 (redundant case) usually involves the division of the n variables into n-6 controls, and six compensators. Loop closure is achieved for arbitrary values of the controls by solving the inverse kinematic problem for the six compensators. These methods can be used to explore conformational variability of loops with more than six variables [7] and have also been used to generate whole-protein conformational ensembles [8]. Linear inverse kinematics can model only small changes in conformation while maintaining loop closure. For that reason, the linear method, applied iteratively, is ideally suited to modelling protein loop dynamics and has been applied to determine the nature of self-motions in protein loops [9,10].

One of the issues addressed in this study is whether all the conformations generated using nonlinear inverse kinematics methods for the redundant case are dynamically accessible to each other.

### 1.3. β-turns

If not all, then almost all globular proteins have β-turns as they bring about the abrupt changes in the path of the protein chain required to create a globular structure. β-turns are normally defined by having a hydrogen bond between residue *i* and residue *i* + 3, and the type is defined by the ϕ , ψ angles of the intervening residues *i* + 1 and *i* + 2. Venkatachalam [11] was the first to classify β-turns, followed later by Lewis [12], Richardson [13] and Wilmot & Thornton [14]. The number of different types of β-turns depends on the classification method used and in more recent studies some have been discarded and new ones introduced [15,16]. In this study, we will concentrate on types: I, I′, II and II′ which represent the vast majority of β-turns in proteins.

Although the results presented are for β-turns, the findings of this study can be generalized to any protein segment and have implications for both protein native state dynamics and protein folding.

## 2. Methods

This work uses a linear kinematics method that writes down the relative translation and rotation of the end group of a protein main-chain segment owing to variation of its internal coordinates. Here, we keep all those degrees of freedom fixed that are relatively constrained compared with the variation of the ϕ , ψ angles. This means all bond lengths, bond angles and ω -torsion angles are kept at their initial values. The segments here are defined from the C_α_ on residue i to the C_α_ on residue i+N . The internal variables of which there are, n=2N , can be expressed as a column vector, $\tau ={\left(\psi_{i}\;\;\phi_{i+1}\;\;\psi_{i+1}\;\phi_{i+2}\;\;\psi_{i+2}\ldots \;\phi_{i+N-1}\;\;\psi_{i+N-1}\;\;\phi_{i+N}\right)}^{t}$ , where t denotes the transpose. The relative position and orientation of the end group (atoms N, C_α_ and C of residue *i + N*) relative to the start group (atoms N, C_α_ and C of residue *i*), referred to here as the ‘end conformation’, is a function of τ . The articles by Hayward & Kitao [9,10] showed a simple way to calculate the 6×n Jacobian matrix, $J\left(\tau \right)$ , for the translational and rotational displacement of the end group relative to the start for a small change in τ , in the linear approximation, as:


(2.1) $$\left(\begin{array}{c} \delta \varphi \\ \delta d \end{array}\right)=\;J\left(\tau \right)\delta \tau ,$$

where δφ and δd are both 3×1 matrices for the relative rotational and translational displacement of the end group relative to the start group, and δτ is a small change in τ . Mainly we will be concerned with a segment five residues in length: that is N=4 and n=8 . In this case, equation (2.1) represents an underdetermined system as there are six equations in eight unknowns.

In this work, two main algorithms were used. The first moves the end group directly to a target location through a rotational and translational displacement, ΔΦ and ΔD . To do this, ∆Φ and ∆D were divided into small increments δφ and δd , and the end moved iteratively by changing the τ angles according to,


(2.2) $$\delta \tau =\;J{\left(\tau \right)}^{+}\left(\begin{array}{c} \delta \varphi \\ \delta d \end{array}\right),$$

where ${J\left(\tau \right)}^{+}$ denotes the pseudo inverse of $J\left(\tau \right)$ . Use of the pseudo inverse means that the solution, δτ , has the smallest Euclidian norm in an underdetermined system. Note for a segment length four residues, where N=3 and n=6 , $J\left(\tau \right)$ will be a square 6×6 matrix and if full rank, the pseudo inverse is the usual inverse. The first algorithm targets the end of the segment.

**Algorithm 1, End Targeting:** Iteratively solve equation (2.2) to move the end by a given rotational and translational displacement, ΔΦ and ΔD .

The other main algorithm randomly moves the configuration point on the null space of $J\left(\tau \right)$ , by iteratively solving:


(2.3) J(τ)δτ=0.

Motion in the null space does not change the end conformation. As described previously [10], the δτ are small steps in random directions determined by randomly combining the orthonormal set of null space vectors of $J\left(\tau \right)$ , which are tangents to the nonlinear self-motion manifold. If occasional drift of the end group occurs, a minimally disruptive reset is performed [10]. Random exploration maintaining the end conformation is a self-motion.

**Algorithm 2, Self-Motion:** Iteratively solve equation (2.3), selecting random directions in the null space at each step to undergo a random self-motion.

An important theorem for this study is the rank-nullity theorem which for the Jacobian used here means:


(2.4) rank( J(τ))+nullity( J(τ))=n,

where nullity refers to the dimension of the null space—which is also referred to as the degree of redundancy in robotics. This means that if rank( J(τ))=6 , that is $J\left(\tau \right)$ is full rank, then nullity( J(τ))=n−6 . For a four-residue segment, where n=6 this means the null space is of dimension zero and so with fixed ends the configuration of a four-residue segment is also fixed. This is the non-redundant case. For a five-residue segment, where n=8 , the null space is a two-dimensional subspace in the full eight-dimensional configuration space. This is then equivalent to an 8R redundant manipulator. Rank and nullity can be monitored by performing singular value decomposition (SVD). SVD decomposes a matrix into a product of three matrices:


(2.5) $$J\left(\tau \right)=USV^{t},$$

where U is a 6×6 orthogonal matrix, V is an n×n orthogonal matrix and S is a 6×n rectangular diagonal matrix of singular values, $s_{11}$ , $s_{22}$ , …, $s_{66}$ . If $J\left(\tau \right)$ is of full rank 6, the singular values are all non-zero, the first six columns of V span the row space of $J\left(\tau \right)$ and the final n-6 columns of V span the null space. At a singular point where $s_{66}=0$ , all other singular values remaining non-zero, rank( J(τ))=5 , and for a five-residue segment, the null space dimension increases from 2 to 3.

To help explain the topological concepts that arise in this work it will be useful to consider the 2R and 3R planar manipulators from the field of robotics.

### 2.1. 2R and 3R planar manipulators

Before dealing with the 3R planar manipulator, consider the 2R planar manipulator. The Jacobian is $J\left(\theta_{1},\theta_{2}\right)$ , a 2×2 matrix that relates changes in the two joint angles, one for the first link, $\theta_{1}$ , the other for the second link, $\theta_{2}$ , to changes to the x , y coordinates of the end of the second link, i.e. the end-effector. This is a non-redundant manipulator (the end-effector has two degrees of freedom) and the general solution for the linear approximation that relates joint angle changes to displacement of the end is:


(2.6) $$\left(\begin{array}{c} \delta \theta_{1}\\ \delta \theta_{2} \end{array}\right)=J{\left(\theta_{1},\theta_{2}\right)}^{-1}\left(\begin{array}{c} \delta x\\ \delta y \end{array}\right).$$

For the arm-up, /\, and arm-down, \/, configurations, $rank\left(\;J\left(\theta_{1},\theta_{2}\right)\right)=2$ and $nullity\left(J\left(\theta_{1},\theta_{2}\right)\right)=0$ , and when the end is fixed, so is the configuration. The arm-up and arm-down configurations can only interconvert via the configurations, where $\theta_{2}=180{}^{\circ}$ or $\theta_{2}=0{}^{\circ}$ . At these configurations $J\left(\theta_{1},\theta_{2}\right)$ is singular, the inverse does not exist, $rank\left(\;J\left(\theta_{1},\theta_{2}\right)\right)=1$ and $nullity\left(J\left(\theta_{1},\theta_{2}\right)\right)=1$ . In other words, at the singularity, the null space dimension increases from 0 to 1 and the manipulator can move through the singular configuration from the set of arm-up configurations to the set of arm-down configurations, or vice-versa. Note for each arm-up configuration, there is a corresponding arm-down configuration that is its mirror image.

**Figure 1.**
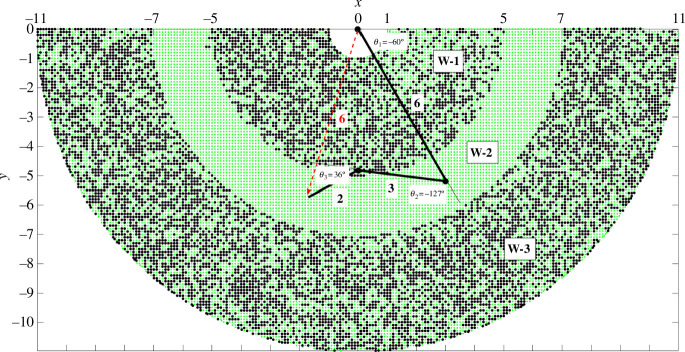
3R planar manipulator workspace. The link lengths are 6, 3 and 2. The starting configuration of the arm has angles 𝜃_1_ = −60°, 𝜃_2_ = −127°, 𝜃_3_ = 36°. The green dots are located at positions that can be reached by the end (end of final link) from the starting configuration. Regions in white cannot be reached. The black dots are at positions where, after targeting the end to this position followed by self-motion (fixed-end exploration) and then return of end to its original position, a mirror configuration of any of the self-motion configurations of the starting configuration occurs—identified by having a positive 𝜃_2_ angle. A boundary between a green and black region, called a ‘critical value manifold’ or ‘Jacobian surface’, divides the workspace into three ’W-sheets’, W-1, W-2 and W-3.

**Figure 2.**
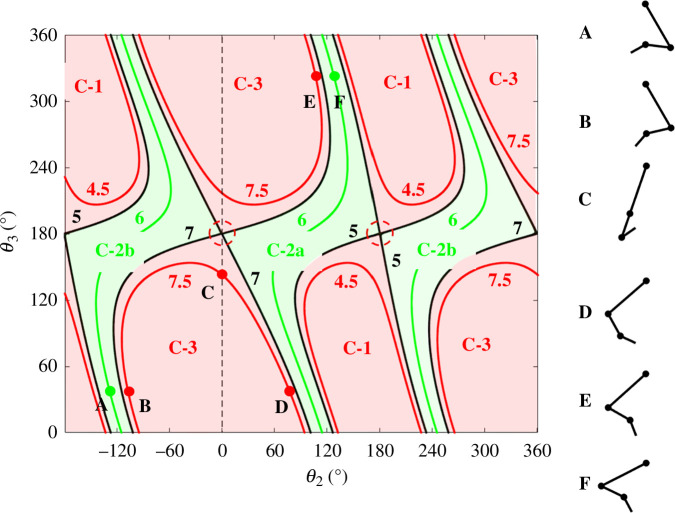
Configuration space of the 3R planar manipulator in figure 1, with link lengths 6, 3 and 2. *θ*_1_ is not shown as results do not vary with *θ*_1_. The three W-sheets are pre-mapped into four regions in the configuration space called ‘C-bundles’: C-1, C-2a, C-2b and C-3, coloured light red, light green, light green and light red, respectively. Each line corresponds to a self-motion, the number in the colour of the line giving the end distance. The black lines indicate co-regular surfaces which demarcate the C-bundles. The red broken circles indicate a singular point on the co-regular surface. The W-2 region pre-maps to two C-bundles, C-2a and C-2b. To go from C-2a to C-2b, one must cross either one of the two co-regular surfaces at end distances 5 or 7. The manipulator configurations A–F illustrate an example. To go from A in C-2b at end distance 6, to its mirror configuration F in C-2a, one can move across the co-regular surface to B by extending the end to 7.5, then undergo a self-motion to E through C and D, and then finally contract the end back to 6 to reach F.

**Figure 3.**
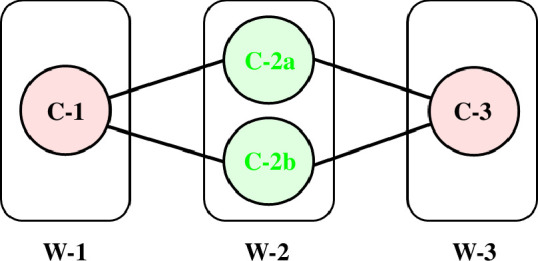
Connectivity map for the 3R planar manipulator in figure 1. The rectangles refer to the W-sheets in figure 1 and the circles refer to the C-bundles in figure 2. To go from C-2a to C-2b one must follow the edges, through either C-1 or C-3.

The 3R planar manipulator has an extra link and with it an extra joint and joint angle, $\theta_{3}$ . The 3R planar manipulator is a redundant manipulator and for a general end position $nullity\left(J\left(\theta_{1},\theta_{2},\theta_{3}\right)\right)=1$ . This means that when the end of the third link is fixed the configuration can change by moving in the one-dimensional null space of $J\left(\theta_{1},\theta_{2},\theta_{3}\right)$ , i.e. undergo a self-motion. Figure 1 shows the workspace of the 3R planar manipulator with link lengths, 6, 3 and 2, an example taken from Lück & Lee [17]. The workspace is the region that can be reached by the end of the third link. The workspace is divided into three regions called W-sheets: W-1, W-2 and W-3. Figure 2 shows the configuration space for $\theta_{2}$ and $\theta_{3}$ . The configuration space is often referred to as a ‘pre-map’ of the workspace. The lines in figure 2 are traces of one-dimensional self-motions at given end distances. The green lines show self-motions with the end in W-2 (end distance of 6), red lines self-motions with the end in W-1 (end distance of 4.5) or W-3 (end distance of 7.5), and the black lines self-motions with the end on the W-1/W-2 boundary (end distance of 5) or the W-2/W-3 boundary (end distance of 7). Indicated by the red broken circles are the singular points where, $\theta_{2}=0{}^{\circ}$ , $\theta_{3}=180{}^{\circ}$ and $\theta_{2}=180{}^{\circ}$ , $\theta_{3}=180{}^{\circ}$ . Self-motions that go through singular points, where $nullity\left(J\left(\theta_{1},\theta_{2},\theta_{3}\right)\right)=2$ , are called ‘co-regular’ self-motions. The traces of the self-motions at end distances within the W-sheets are homeomorphic to circles, whereas the co-regular self-motions at the W-sheet boundaries are homeomorphic to a figure ‘8’ with the singular point at the ‘waist’ of the 8 [1], where the null space dimension increases from 1 to 2. A W-sheet boundary is called a ‘critical value manifold’ or ‘Jacobian surface’ and the pre-map of a critical value manifold which combines all the traces of corresponding co-regular self-motions is called a ‘co-regular surface’. The co-regular surfaces divide the configuration space into different regions called ‘C-bundles’ as indicated in figure 2. There are four C-bundles, C-1, C-2a, C-2b and C-3, which correspond to W-sheets, W-1, W-2, W-2 and W-3, respectively. Figure 3 shows a ‘connectivity map’ for the 3R planar manipulator as given in Lück & Lee [17] that summarizes the relationship between C-bundles and W-sheets.

In contrast to the non-redundant 2R planar manipulator, where to go from one configuration to its corresponding mirror image one must go through a singular configuration, in a redundant manipulator one can avoid a singular configuration. For the redundant 3R planar manipulator, to go from configuration A in C-2b in figure 2 to its mirror image configuration F in C-2a, the following steps avoid a singular configuration:

— **step 1:** extend the end from A in C-2b to conformation B in C-3, thus crossing a co-regular surface;
— **step 2:** perform a self-motion in C-3 from B to E through C and D; and
— **step 3:** return end to its original position by moving across the co-regular surface from E to F in C-2a.

Note that configuration F is the mirror image of A. More generally, for every configuration in C-2b, a mirror image configuration exists in C-2a.

For those not familiar with inverse kinematics in robotics, an excellent introduction to robotic manipulators is given in the book by Lynch & Park [18], which describes inverse-kinematics methods.

Calculations were carried out using Matlab version: 9.11.0.1837725 (R2021b) Update 2. Matlab uses SVD (command: *svd*) to calculate both the pseudo inverse (command: *pinv*) and the null space (command: *null*) of a matrix.

Table 1 gives the parameter value settings used throughout this work for both algorithms.

## 3. Results

### 3.1. Type I turn: random structure returns versus self-motion

**Figure 4.**
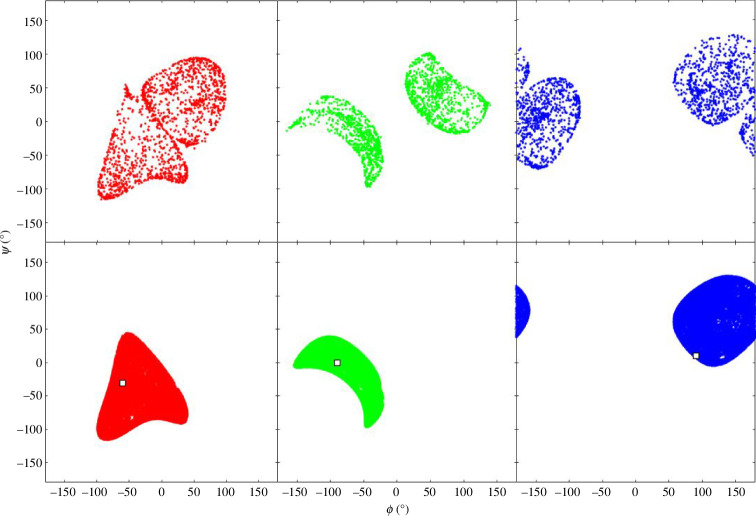
Ramachandran plots for type I turn from the experimental structure, PDB: 2BK9, chain A, 147–151. Asp148 (red), Asp149 (green) and Gly150 (blue). Top row: result of bringing the end of a structure with randomly generated ϕ,ψ angles (5000 examples) to the same end conformation as the type I turn experimental structure showing the emergence of the type I´ turn. Bottom row: result of self-motion (300 000 steps) starting from type I turn experimental structure. The squares show the ϕ,ψ angles of the experimental structure. Note these are projections of a two-dimensional surface in an eight-dimensional space.

**Figure 5.**
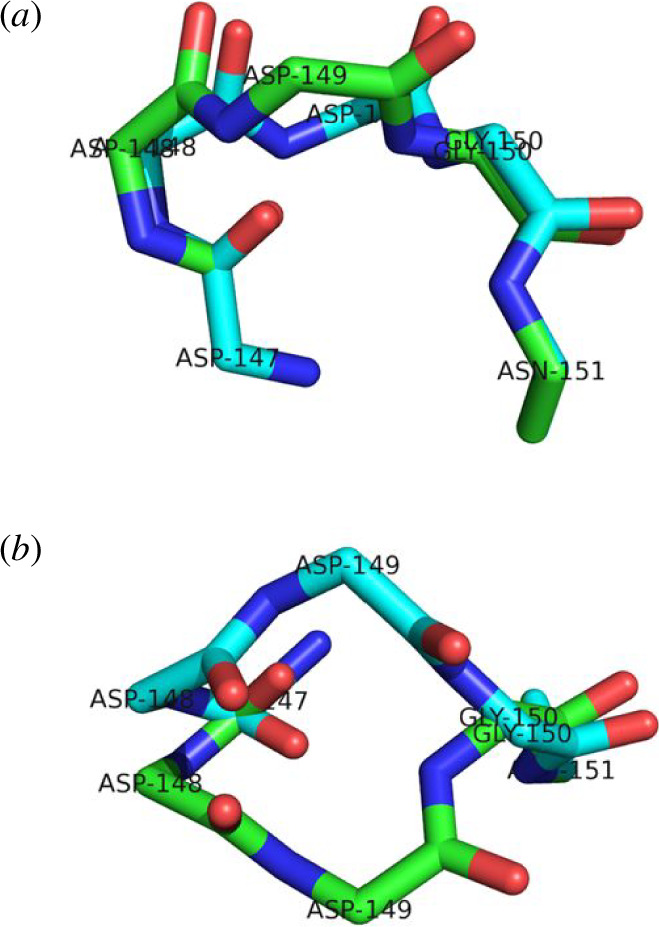
In green the type I turn from PDB structure: 2BK9 chain A, residues 147–151. In cyan the type I´, after targeting the ends of structures with randomly generated ϕ,ψ angles to the end conformation of the type I turn. See text for details. (*a*) Side view. (*b*) Top view.

Algorithms 1 and 2 were used on the type I turn. The experimental structure of the type I turn was taken from the article by de Brevern [15]: Protein Data Bank (PDB) structure 2BK9, chain A, residues 147–151. Starting with the four-residue segments 147–150 and 148–151, structures were generated by selecting random ϕ , ψ angles. For both segments, the ends of the randomly generated structures were targeted back to the end conformation of the experimental structure using algorithm 1 . For both, all the resulting structures were a type I turn. This process was repeated for the five-residue segment, 147–151. The result in a Ramachandran plot is shown in the top row of figure 4. Starting from the type I turn experimental structure, self-motions were carried out using algorithm 2 . The bottom row of figure 4 shows the result. As can be seen by comparing the top and bottom rows of figure 4, the randomly generated structures with the end conformation of the type I turn create a new structure that is dynamically inaccessible to the original type I turn. This new structure is consistent with a type I′ turn, the enantiomeric counterpart of the type I turn, i.e. its mirror structure. Figure 5 shows two structures, the experimental type I turn and a type I′ turn that resulted from this process. The analogy with the 3R planar manipulator, where two mirror configurations of the arm, one in C-2a, the other in C-2b, suggests the same principle applies here and that the two enantiomeric turn structures are from different C-bundles in the same W-sheet. From a protein folding perspective, it suggests that as the ends come together a W-sheet boundary is crossed, and the structure will be locked into either becoming a type I or a type I′ turn.

**Table 1.** Parameter values for algorithms 1 and 2.

parameter description	value
step size factor for moving end: δφ/∆Φ and δd/∆D	0.001
rmsd^a^ threshold on end group (N, C_α_ and C atoms of final residue) for successful targeting of end^b^	0.1 Å
step size for self-motion, (null space exploration), $\left|\delta \tau \right|$	2°
msd^c^ threshold on end group in self-motion for end reset	0.001 Å^2^

^a^ rmsd: root-msd

^b^ Structures that did not satisfy this threshold were discarded.

^c^ msd: mean-square deviation.

### 3.2. Types I and I′ turns belong to different C-bundles in the same W-sheet

**Figure 6.**
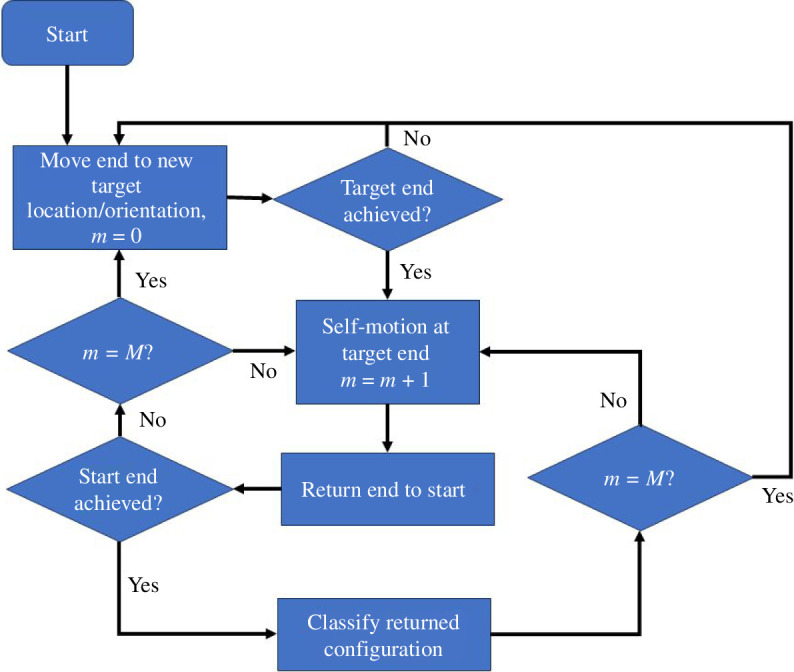
Flow chart for the algorithm used to discover W-sheet boundaries. The end is moved using algorithm 1 and self-motions generated with algorithm 2. 𝑀 is the total number of phases of self-motion attempted. The starting configuration of self-motion 𝑚 was the final configuration of self-motion 𝑚 ― 1. The nature of the returned configurations determines whether a W-sheet boundary has been crossed.

In the case of the 3R planar manipulator, analytical methods can be used to determine W-sheets and co-regular surfaces. As this did not seem feasible for proteins, the process described in figure 6 was used. The idea behind this process can be understood by considering **steps 1–3** for the 3R planar manipulator (see §2). If in **step 1** the extension of the end does not take it across the W-sheet boundary, then a self-motion followed by a return of the end to its original position would only result in another C-2b configuration. Only when the extension in **step 1** takes the end beyond the W-sheet boundary, is it possible that after a self-motion and return of the end, a C-2a configuration will occur. Carrying out this process systematically for different extensions it should be possible to find the W-sheet boundary. To verify that the process worked it was first applied to the 3R planar manipulator with link lengths, 6, 3 and 2, to establish whether it could reproduce the known results as given in Lück & Lee [17]. From the starting configuration with angles $\theta_{1}=-60{}^{\circ}$ , $\theta_{2}=-127{}^{\circ}$ and $\theta_{3}=36{}^{\circ}$ , the end was systematically targeted to an array of points in the intervals x:[-11,11] , y:[0,-11] , self-motions were carried out and the end returned to the starting configuration end position. The returned structure either belonged to C-2b (the C-bundle of the starting configuration) or C-2a, as judged by the range of $\theta_{2}$ . Figure 1 confirms that the process in figure 6 works.

**Figure 7.**
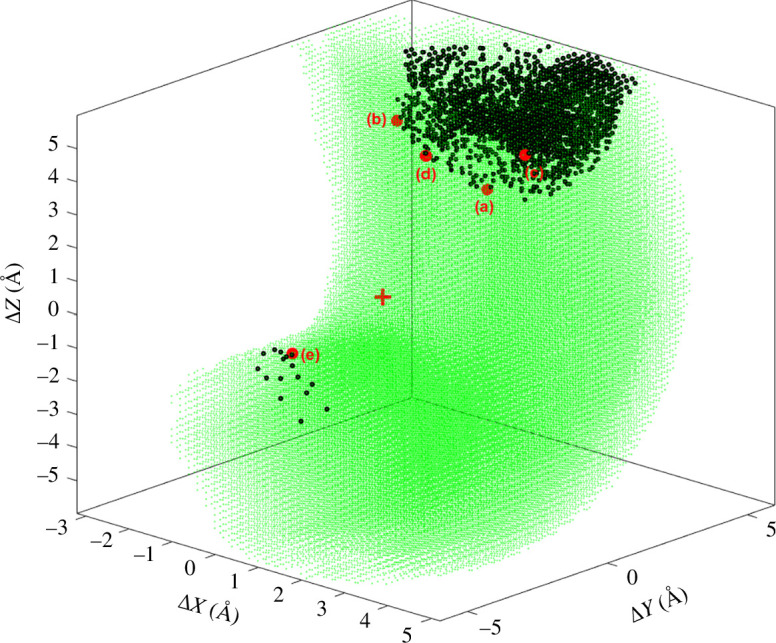
Analogous to figure 1 for the type I turn. The process used to create the figure is described in figure 6. The end was systematically targeted to displacements (without rotation) in the intervals Δ*X*:[− 6,6], Δ*Y*:[−6,6], Δ*Z*:[−6,6] in 0.2 Å steps. Green dots indicate achievable displacements of the end residue from its starting point in the type I turn. The red cross is at (0,0,0) so represents the type I/I′ turn end residue position. Regions in white outside the green region cannot be reached. The black dots are at displacements of the end residue where, after targeting the end residue to this position followed by a self-motion and then return of end residue to its original position, a type I′ turn occurs as identified by Asp149 having a positive ϕ angle. The boundary between the green and black regions is a W-sheet boundary that divides the workspace into W-sheets. Labelled red points are samples on the W-sheet boundary.

**Figure 8.**
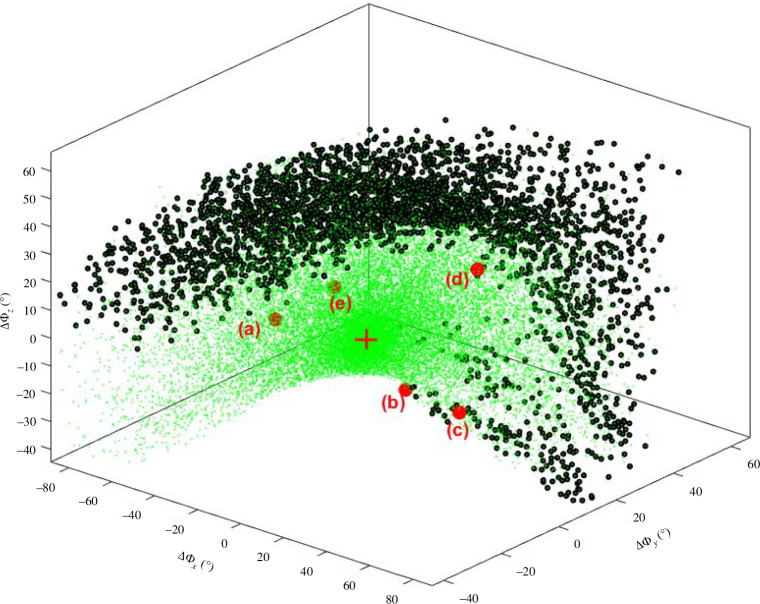
As for figure 7 but for 100 000 random rotations (no translation) of the end residue, Asn151.

For the type I turn the workspace is six-dimensional and too large to explore exhaustively. As this is a proof-of-principle study, it is sufficient to demonstrate it for the following two workspace subspaces: one where the end undergoes translation and no rotation, the other where the end undergoes rotation and no translation. For the case of pure translation, the method used for the 3R planar manipulator was used. The end was systematically targeted to displacements in the intervals ΔX:[-6,6] , ΔY:[-6,6] , ΔZ:[-6,6] in 0.2 Å steps. At each end position the process in figure 6 was carried out, which comprised 20 rounds ( M=20 ) of 5000 × rand-step self-motion, where ‘rand’ is a random number between 0 and 1 on a uniform random distribution. Figure 7 which is analogous to figure 1 shows the result. As a further check, this process was repeated starting from the type I′ turn shown in figure 5. As expected, the same W-sheet boundary was found. The minimum value for $\left|\Delta D\right|$ on the W-sheet boundary is 3.8 Å. It is noted that movement of the end in certain directions, does not result in the W-sheet boundary being crossed. For these directions, the configuration is locked into the type I or type I′ turn depending on its starting configuration. Figure 8 shows the result for pure rotation of the end residue. In this case, 100 000 random rotations of the end were generated. At each end orientation, the process in figure 6 was carried out, which comprised five rounds ( M=5 ) of 1000 × rand-step self-motion. Again, a W-sheet boundary was discovered. The minimum value for $\left|∆\Phi \right|$ on the W-sheet boundary is 26.7°.

**Figure 9.**
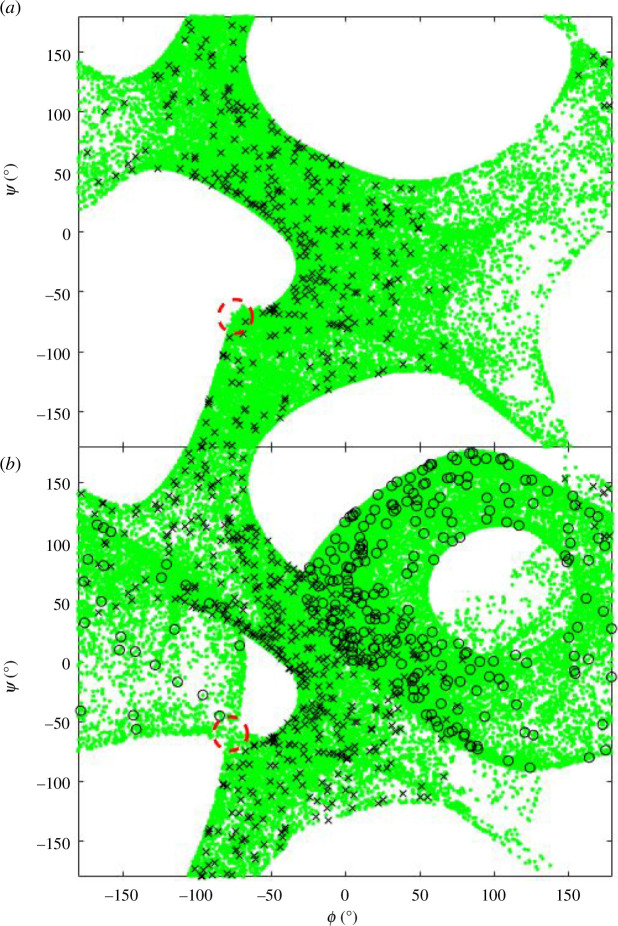
Ramachandran plots for ϕ,ψ angles of Asp149 in self-motions for type I turn. Green dots are sample points on self-motion manifolds. Crosses show conformations that return to type I turns and black open circles conformations that return to type I′ turns. (*a*) With end conformation just before the W-sheet boundary at point (a) in figure 7. (*b*) With end conformation exactly on point (a) on the W-sheet boundary and thus showing a co-regular self-motion. The broken red circles indicate a point close to and at a singular point on the co-regular surface. Column (a) in table 2 gives the 𝜙, 𝜓 angles of all residues at the singular point.

**Figure 10.**
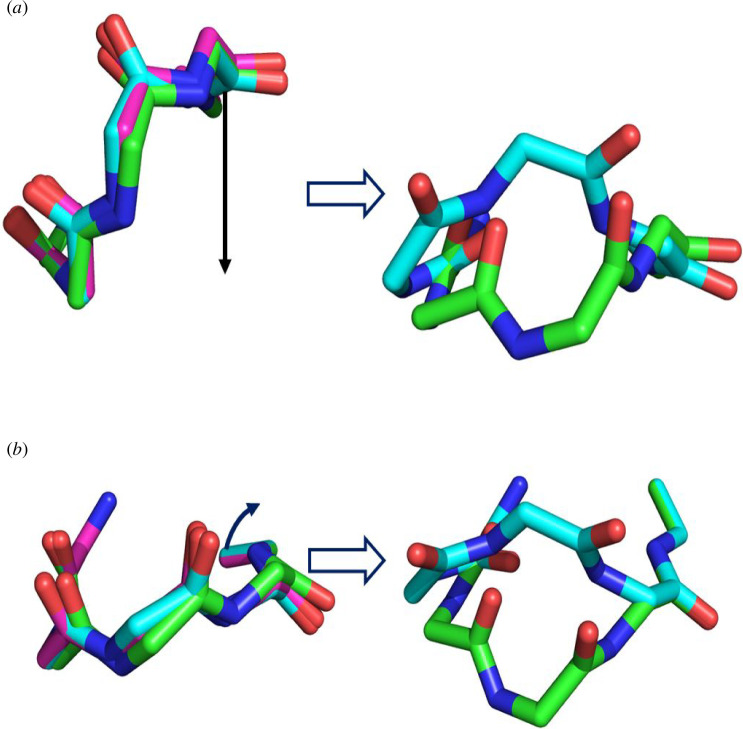
(*a*) In magenta, main-chain structure at singular point on the co-regular surface in figure 9 corresponding to point (a) in figure 7. Table 2 gives the 𝜙, 𝜓 angles for this singular structure. In green, main-chain structure that is close to the singular structure and which results in a type I turn when the end is translated (indicated by downward pointing arrow) to its position in the type I/type I′ turn. In cyan, main-chain structure close to singular structure which results in a type I′ turn. (*b*) Analogous to (*a*) for pure rotation of the end. The magenta structure is at the singular point on the co-regular surface corresponding to point (d) in figure 8. Table 3 gives the 𝜙, 𝜓 angles for the singular structure. The pure rotation (indicated by curved arrow) of the end to its orientation in the type I/type I′ turn brings the structures on either side of the singular structure to the respective type I and type I′ turns.

Five points were selected at different locations on the W-sheet boundary of both workspace subspaces to confirm the presence of a co-regular surface and to investigate the nature of the singular configurations. Figure 9 shows self-motion trajectories with the end located just before, and exactly on the W-sheet boundary at position (a) in figure 7. For the end located on the W-sheet boundary, the self-motion has all the characteristics of a co-regular self-motion with a new region ‘bursting out’ from the original region where the end is located just before the boundary. In the original region, all configurations become type I turns when the end was returned, whereas in the new region all configurations became type I′ turns. The point where the new region bursts from is a singular point where the null space dimension increases from 2 to 3. This is equivalent to the ‘waist’ of the figure ‘8’ for the 3R planar manipulator. For two structures on the co-regular surface ‘either side’ but very close to the structure corresponding to the singular point in figure 9, figure 10*a* shows the structural transitions that occur when the end is translated back to the type I/I′ end conformation; one results in a type I turn, the other in a type I′ turn. Figure 10*b* shows the equivalent when the end is rotated back to the type I/I′ end orientation from W-sheet boundary position (d) in figure 8.

**Table 2.** *ϕ*, *ψ* and τ (pseudo torsion) angles of singular structures at translated end conformations in figure 7. (Δ*X*, Δ*Y*, Δ*Z*: (a) 0.76, 2.54, 2.80 (b) −1.0, 2.1, 4.3 (c) 2.5, 1.2, 4.9 (d) 0.6, 0.6, 4.3, (e) 1.4, −5.3, 0.4; units are Å.)

end position label:	(a)	(b)	(c)	(d)	(e)
$\psi_{147}$	−93.3°	−102.1°	−129.1°	−109.0°	19.7°
$\phi_{148}$	−51.3°	−28.8°	−19.7°	−31.7°	−12.3°
$\psi_{148}$	−125.2°	−59.7°	−173.5°	151.6°	−175.4°
$\phi_{149}$	−78.2°	−153.6°	−10.0°	−10.2°	−0.5°
$\psi_{149}$	−59.1°	−8.4°	−170.7°	4.0°	67.0°
$\phi_{150}$	−133.9°	−172.5°	−40.3°	−179.0°	132.6°
$\psi_{150}$	78.6°	84.9°	27.6°	67.3°	62.1°
$\phi_{151}$	−128.5°	−134.1°	−57.4°	−125.2°	42.6°
$\tau_{148-149}$	−44.5°	−41.2°	−9.9°	−49.2°	18.5°
$\tau_{149-150}$	7.1°	23.1°	−27.7°	25.7°	33.0°

**Table 3.** *ϕ*, *ψ* and τ (pseudo torsion) angles of singular structures at rotated end conformations in figure 8. (Δ*Φ_x_*, Δ*Φ_y_*, Δ*Φ_z_*: (a) −25.2°, −13.2°, 7.8° (b) −28.9°, 34.9°, −41.8° (c) 36.1°, 5.0°, −19.1° (d) 48.3°,2.1°,36.7°, (e) −70.8°, 41.7°, −18.8°.)

end position label:	(a)	(b)	(c)	(d)	(e)
$\psi_{147}$	−165.4°	55.0°	63.6°	−147.7°	88.2°
$\phi_{148}$	−2.6°	−9.8°	−16.4°	−37.8°	−22.0°
$\psi_{148}$	−57.4°	43.6°	69.1°	−98.6°	118.9°
$\phi_{149}$	−178.0°	178.7°	146.0°	−103.5°	75.2°
$\psi_{149}$	2.8°	−2.1°	0.6°	−48.4°	38.7°
$\phi_{150}$	−178.1°	−179.5°	179.4°	−140.1°	149.9°
$\psi_{150}$	55.9°	51.0°	62.8°	79.8°	119.2°
$\phi_{151}$	−134.8°	160.1°	72.9°	131.6°	132.8°
$\tau_{148-149}$	−55.2°	39.0°	26.8°	−38.8°	15.4°
$\tau_{149-150}$	26.9°	21.0°	39.6°	12.7°	37.2°

For all five points (a)–(e) on figure 7 and the five points (a)–(e) on figure 8, the ϕ , ψ angles at the singular points on the corresponding co-regular self-motions were determined. SVD was used to confirm they were singular by checking that $s_{66}=0$ . Tables 2 and 3 give the ϕ , ψ angles of these singular structures for the pure translation and pure rotation workspace subspaces, respectively. Although in the majority of cases, at least one of the ϕ , ψ angles is either close to 0° or 180°, this is not always the case, e.g. cases (a) and (d) in tables 2 and 3, respectively. Also given in tables 2 and 3, are the pseudo torsion angles, $\tau_{148-149}$ (formed by $C_{147}^{\alpha }-C_{148}^{\alpha }-C_{149}^{\alpha }-C_{150}^{\alpha }$ ) and $\tau_{149-150}$ (formed by $C_{148}^{\alpha }-C_{149}^{\alpha }-C_{150}^{\alpha }-C_{151}^{\alpha }$ ) of the singular structures. The values for these angles in the type I and type I′ turns are 48.2°, −91.7° and −61.1°, 60.9°, respectively. As one might expect, the pseudo torsion angles at the singular points suggest flatter structures, with both angles in the interval [−60°, 60°] and at least one of them restricted to the interval [−30°, 30°]. It is important to realize, however, in a folding process the conformations need not go through a singular configuration.

### 3.3. α-helix, β-strand and type II turn: random structure returns versus self-motion

**Figure 11.**
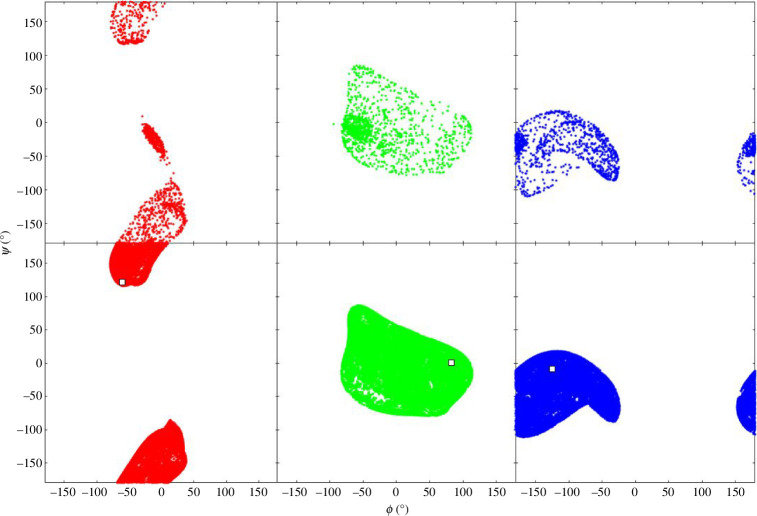
Ramachandran plots for type II turn from 1H16, chain A, 424–428. Val425 (red), Gly426 (green) and Lys427 (blue). Top row: results of bringing the ends of the structure with randomly generated 𝜙, 𝜓 angles to the same end conformation as the type II turn. Bottom row: result of self-motion (fixed-end exploration) starting from type II turn. The squares show the 𝜙, 𝜓 angles of the starting structure.

Figure 11 shows the equivalent Ramachandran plot to figure 4 for the type II turn. The turn was also taken from the article by de Brevern [15]: PDB structure 1H16, chain A, residues 424–428. It does not show the appearance of a type II′ turn, although comparing the random returns to the self-motion trajectory for Val425, it is evident that the self-motion is very close to being on a co-regular surface.

The same process was carried out on a five-residue α-helix structure and a five-residue β-strand structure with ϕ , ψ angles taken from Hovmoller *et al*. [19]. No difference was found between the random returns and self-motions for both structures as can be seen in electronic supplementary material, figures S1 and S2. This shows that for both there is only one C-bundle with their end conformation. It shows that when the end-conformation is reached the α-helix and β-strand structures are contained within their respective self-motions.

### 3.4. Type II turn as topologically equivalent to the type I′ turn

**Figure 12.**
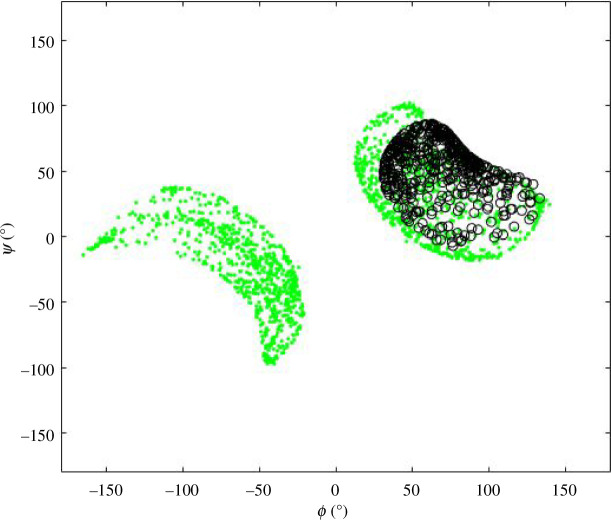
In green is the Ramachandran plot for 𝜙, 𝜓 angles of Asp149 for type I turn end returns from randomly generated 𝜙, 𝜓 angles as shown in figure 4. The black circles are for the equivalent residue to Asp149 in the type II turn, Gly426, which shows that the result of targeting the end conformation of the type II turn to the end conformation of type I/type I′ turn, is the type I′ turn only. The difference between the type I′ distributions is owing to the differences in the values of the bond lengths, bond angles and *ω* torsion angles in the type I and type II turn experimental structures. See the main text for further explanation.

**Figure 13.**
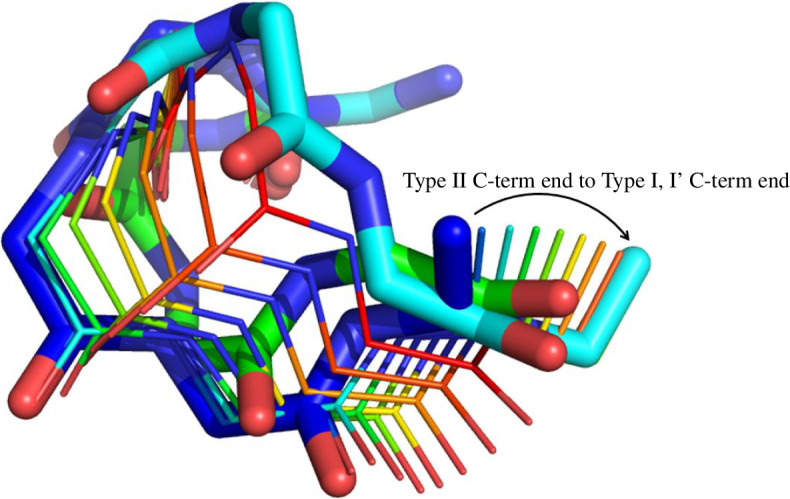
Type II turn in thick blue stick, type I turn in thick green stick, and type I′ turn in thick cyan stick. The thin stick depiction shows the conformations that occur when the end conformation of the type II turn is moved directly (without any self-motion) to the type I/type I′ turn end conformation. As can be seen, the structures (blue to red) move towards the type I′ turn conformation not the type I turn conformation.

**Figure 14.**
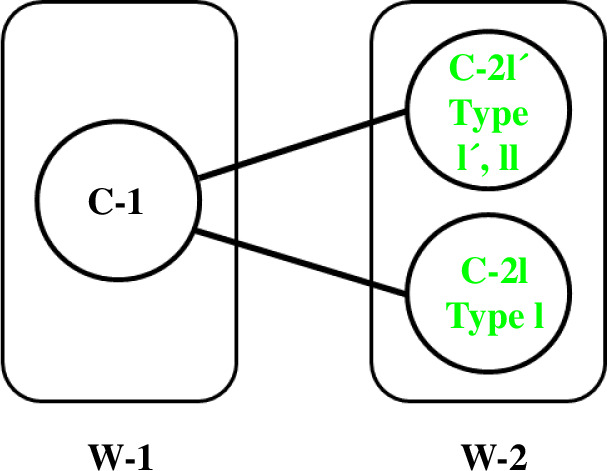
Connectivity map for type I, type I′ and type II turns. Type I′ and type II turns belong to the same C-bundle (C-2I′), but the type I turn belongs to a different C-bundle (C-2I). To go from type I′ or type II turns to a type I turn one must go via C-1 in W-1, i.e. the end must move beyond the W-1/W-2 sheet boundary.

For the type I turn we have discovered three C-bundles in two W-sheets, one for the type I turn (which we name ‘C-2I’ in ‘W-2’), one for the type I′ turn (‘C-2I′’ in ‘W-2’) and one that comprises all configurations with end conformations beyond the W-sheet boundary (‘C-1’ in ‘W-1’). To which of these three C-bundles does the type II belong? To answer this, the end of the type II turn was targeted to the end of the type I/I′ turn. After an initial 1000 × rand-step self-motion, the end was rotated and translated in 10 equal incremental movements to reach the type I/I′ turn end conformation, each incremental movement being followed by a 1000 × rand-step self-motion. This whole procedure was repeated 500 times and the ϕ , ψ angles at the end of the final phase of self-motion plotted in figure 12 showing that all resulting structures are type I′ turns. Thus, the type II turn is in the same C-bundle as the type I′ turn and is therefore topologically equivalent to the type I′ turn, not the type I turn. Figure 13 shows intervening conformations when the end is directly moved from the type II end to the type I/I′ turn end conformation showing the transition from the type II to type I′ turn. The findings can be summarized in the connectivity map of figure 14, which is only a small part of the connectivity map for the five-residue segment which in its entirety would also include the α-helix, β-strand structures and any other structure from a distinct C-bundle.

It was found that direct targeting to the type I/I′ turn end conformation from five-residue α-helix and β-strand structures was impossible, probably owing to the path intersecting a singularity.

## 4. Discussion

It has been shown using the five-residue main-chain segment with its ϕ , ψ torsions as the only degrees of freedom, that concepts regarding self-motions in redundant robotic manipulators [1,17] also apply to protein segments. How would this help in the understanding of protein folding and protein dynamics?

### 4.1. Role in protein folding

In protein folding, a compactification of the protein chain occurs in a process that is energetically driven by hydrophobic regions creating an interior where they are shielded from the water solvent. The appearance of turns, whether they are β-turns or the 4-turns of the α-helix, is a feature of protein folding as they bring about the abrupt changes in chain direction necessary for a compact folded structure. Consider the five-residue segment early in the folding process and in a C-1 configuration in W-1. Consider also that during the folding process its ends, initially well separated, move progressively towards the end conformation of the type I/I′ turn. As the end conformation comes close to the W-1/W-2 boundary and the configuration is close to the co-regular surface separating C-1 and C-2I/C-2I′, its self-motion effectively combines the configurational spaces of C-2I and C-2I′. As the W-1/W-2 boundary is crossed, it will go to either C-2I or C-2I′ depending on its configuration when crossing the co-regular surface. It is reasonable to suggest that the non-bonded interactions determine this configuration and select the turn type that results. In terms of the connectivity map in figure 14, these interactions select the edge, C-1 to C-2I or C-1 to C-2I′, as one goes from W-1 to W-2. In this model, there are two drivers determining structure during folding, the first determines the end conformations of segments, and the second selects the configuration determining the turn type that results as a co-regular surface is crossed. Crossing a co-regular surface will result in an abrupt change in configurational entropy. In the case of the type I/I′ turn, there will be a dramatic reduction in configurational entropy in going from C-1 bundle to the C-2I or C-2I′ bundle (see figure 9). Significantly, during folding, intermediate configurations, in either C-2I or C-2I′, will be locked into their native structures before their ends reach their final conformation. Such a process suggests a link to the folding funnel concept [2].

### 4.2. Role in protein dynamics

In an earlier study [9], it was noticed that for a five-residue loop with fixed ends and its $\phi_{i+1}$ angle constrained (in this case self-motions have one degree of freedom and their trajectories can be visualized as lines in a Ramachandran plot), a so-called ‘looping-out’ occurred in the self-motion trajectory for only a very small change in the constrained $\phi_{i+1}$ angle. Although it was not realized at the time, this ‘looping-out’ is analogous to what is seen in figure 9, and was the emergence of a new region on a co-regular surface. The change in the $\phi_{i+1}$ angle has the same effect as a change in the end position here. This means that the topological concepts regarding self-motions also apply to protein loop dynamics in native proteins and consequently their complex dynamics could be characterized in connectivity maps.

As mentioned above, both linear and nonlinear inverse-kinematics methods have been applied in protein research. This study suggests that application of nonlinear inverse kinematics methods for the study of loop dynamics might result in solutions that are on different self-motion manifolds and would consequently be dynamically isolated from each other. It seems that some studies which use non-linear inverse-kinematics to model protein loop dynamics do not take this possibility into account.

### 4.3. Influence of other degrees of freedom

How would variations in the harder internal degrees of freedom, such as bond lengths, bond angles and ω -torsion angles affect results? The small effect of these other degrees of freedom in comparison to variation in ϕ , ψ angles can be seen in figure 12. The impact of the hard degrees of freedom on the topological features found here, can best be appreciated by again considering the 3R planar manipulator. If small variations in the lengths of links occur according to a Gaussian probability distribution, then instead of abrupt W-sheet boundaries, W-sheet boundary regions with this probability distribution would result. Consequently, co-regular surfaces would also become regions with associated probabilities. These co-regular regions would still create barriers between C-bundles and the topological features seen here would remain.

### 4.4. Unifying picture of folding and dynamics

Here, we have considered a short protein segment, but would these concepts apply to longer segments? Theorem 1 by Burdick [1] states that for a redundant *n*R robot manipulator, the number of self-motions for a given fixed-end conformation would be no more than the 16 inverse kinematics solutions for the non-redundant 6R manipulator [20,21]. However, this is an upper limit and as *n* increases so the number of distinct self-motions decreases. Indeed, for a flexible string there will be only one self-motion. For a protein, however, the early formation of α-helices during protein folding means that even for long segments, portions of the chain will be effectively rigid, meaning that distinct self-motions should still occur in longer segments.

For a general protein segment, its whole conformational space will be partitioned into distinct non-overlapping regions (C-bundles) demarcated by co-regular surfaces. Self-motions within the same C-bundle are homotopic as the manifold of one can be smoothly deformed into that of another. This means that all self-motions which occur within a C-bundle can be united to produce a set of topologically equivalent motions. Thus, the viewpoint of a self-motion of a segment with fixed ends is superseded by a viewpoint whereby all motions are united, even those that move the ends, as long as the ends do not cross a W-sheet boundary. Self-motions from different C-bundles are not homotopic and there is a dramatic change in the available conformational space as a co-regular surface is crossed. From this perspective, the focus is not on individual conformations but on ensembles, with conformations grouped according to their topological equivalence. In theory, it should be possible to map this entire configuration space. A connectivity map presents a simplified version of this map indicating paths between different C-bundles. A more comprehensive description would also specify W-sheet boundary locations in the six-dimensional space, and their corresponding boundaries (the co-regular surfaces) in the configurational space. For the five-residue segment considered here, such a description would encompass all five-residue conformations found within folded and unfolded proteins.

## Data Availability

The only data used is from the Protein Data Bank (PDB) and can be downloaded from www.pdb.org. The PDB codes for the files used are given in the manuscript. The two main algorithms referred to in the manuscript as algorithm 1 and algorithm 2 are available as electronic supplementary material, 1 and 2, respectively [22]. These are each a single Matlab source code file that can be run as instructed.
